# Neuropathological hints from CSF and serum biomarkers in corticobasal syndrome (CBS): a systematic review

**DOI:** 10.1186/s42466-023-00294-0

**Published:** 2024-01-04

**Authors:** Giulia Remoli, Edoardo Dalmato Schilke, Andrea Magi, Antonio Ancidoni, Giulia Negro, Fulvio Da Re, Maura Frigo, Martina Giordano, Nicola Vanacore, Marco Canevelli, Carlo Ferrarese, Lucio Tremolizzo, Ildebrando Appollonio

**Affiliations:** 1 Neurology Department, Fondazione IRCCS San Gerardi dei Tintori, San Gerardo Hospital, Monza. Via G. Pergolesi, 33, 20900 Monza, Italy; 2 School of Medicine and Surgery and Milan Centre for Neuroscience (NeuroMI), University of Milano-Bicocca, Milano, Italy; 3 National Institute of Health, Roma, Italy; 4 Neurosurgery Unit, Department of Neuroscience, ASST Grande Ospedale Metropolitano Niguarda, Milano, Italy; 5 University of Milan, Milano, Italy; 6 Department of Neuroscience, Sapienza University of Roma, Roma, Italy

**Keywords:** Corticobasal syndrome, Fluid biomarkers, CBS biomarkers, CBS neuropathology, Dementia biomarkers

## Abstract

Corticobasal syndrome (CBS) is a clinical syndrome determined by various underlying neurodegenerative disorders requiring a pathological assessment for a definitive diagnosis. A literature review was performed following the methodology described in the Cochrane Handbook for Systematic Reviews to investigate the additional value of traditional and cutting-edge cerebrospinal fluid (CSF) and serum/plasma biomarkers in profiling CBS. Four databases were screened applying predefined inclusion criteria: (1) recruiting patients with CBS; (2) analyzing CSF/plasma biomarkers in CBS. The review highlights the potential role of the association of fluid biomarkers in diagnostic workup of CBS, since they may contribute to a more accurate diagnosis and patient selection for future disease-modifying agent; for example, future trial designs should consider baseline CSF Neurofilament Light Chains (NfL) or progranulin dosage to stratify treatment arms according to neuropathological substrates, and serum NfL dosage might be used to monitor the evolution of CBS. In this scenario, prospective cohort studies, starting with neurological examination and neuropsychological tests, should be considered to assess the correlations of clinical profiles and various biomarkers.

## Introduction

The term corticobasal syndrome (CBS) describes a rare neurodegenerative disorder characterized by the variable combination of specific cortical and subcortical clinical features (i.e., ideomotor apraxia, sensory neglect, alien limb phenomenon, akinetic-rigid parkinsonism, typically with an asymmetric presentation of limb rigidity, myoclonus, dystonia, or akinesia) and it represents the phenotypic expression of several different underlying pathological processes [1]. Therefore, the term CBS is currently used to describe a clinical syndrome regardless of the underlying pathological process. Corticobasal degeneration (CBD) and Progressive Supranuclear Palsy (PSP) are the two most common neuropathological substrates of CBS, each accounting for about one third of all cases.

CBD is macroscopically characterized by cortical degeneration, often asymmetric, and variable basal ganglia and nigral degeneration, microscopically corresponding to neuronal loss and gliosis, associated with the presence of ballooned achromatic neurons and neuronal and astrocytic thread-like tau inclusions with a cortical distribution. PSP is characterized by tau-enriched tufted astrocytes and neurofibrillary tangles (NFTs) in subcortical nuclei. The third more common neuropathological substrate is Alzheimer Disease (AD) which accounts for—20% of cases, whereas the remaining cases have been variably attributed to Pick’s Disease, Globular Glial Tauopathy (GGT), Anti-IgLON5 disease, Frontotemporal Lobar Degeneration (FTLD) with TDP-43 inclusions (FTLD-TDP) and with fused-in-sarcoma pathology (FTLD-FUS), Lewy Body Disease (DLB), and even Creutzfeldt-Jacob Disease (CJD) [2, 3].

Sporadic presentations represent most CBS cases, but familial cases have been described as well: progranulin gene (GRN) mutation is the most common cause of familial CBS. GRN frontotemporal dementia (GRN-FTD) generally affects the frontal and temporal cortex leading to behavioural changes, executive disfunction, and language disturbances; however, in some cases the parietal cortex and basal ganglia may be affected as well, resulting in parkinsonism and corticobasal syndrome [4], as such, GRN mutation might represent a possible underinvestigated cause of CBS associated with TDP-43 neuropathology. Benussi et al. [5] and Le Ber et al. [6] found a GRN mutation in 11% (1 out of 9 patients) and 3.3% (1 out of 30) of sporadic cases, respectively. Arienti et al. [7] described that GRN may be mutated in almost half of the cases (48%) in genetically determined CBS. Antemortem diagnosis relies on clinical criteria (e.g., Amstrong et al. [8], University of Toronto [9], Mayo Clinic criteria [10], MDS [11], and Cambridge [12]/modified Cambridge criteria [13]) supported, to a limited extent, by ancillary investigations. For instance, asymmetric atrophy and cerebral glucose hypometabolism in the frontoparietal cortex and basal ganglia are typical MRI and FDG-PET findings in CBS patients and a CSF AD profile has been reported in CBS-AD cases. In addition, several studies have reported a significant increase in neurofilament light-chains (NfL) in CBS compared to Parkinson’s disease (PD), PSP, AD and healthy controls [14, 15]. However, studies have yet to investigate the CSF profile of many CBS cases systematically.

Consequently, this biological heterogeneity collects several implications and repercussions: first, in the presence of an atypical clinical presentation, the differential diagnosis with other neurodegenerative disorders based on CSF findings and imaging biomarkers remains challenging, non-specific, and unreliable, raising considerable concerns on optimal patients’ management and counselling.

To now, the clinical complexity of CBS justifies an unstandardised and patient-tailored diagnostic work-up with recurrent identification of “unexpected” radiological or biological features.

Additionally, eventual clinical trials would require diagnostic accuracy for the underlying neurodegenerative processes of CBS to deliver the most suitable disease-modifying agents and patient- tailored interventions. These aspects make CBS a modern challenge for clinicians and a complex pathway for patients and caregivers to walk along.

The present systematic review aimed to investigate the additional value of traditional and cutting-edge CSF and serum/plasma biomarkers in profiling neurodegenerative disorders manifesting with CBS and determine which biomarkers core might be specific and distinctive of CBS.

## Methods

The present systematic literature review was performed following the methodology described in the Cochrane Handbook for Systematic Reviews and was reported based on the PRISMA statement for reporting systematic reviews and meta-analyses [16, 17]. A systematic literature search was conducted in four biomedical databases: (1) PubMed, (2) Cochrane, (3) Scopus, (4) ApaPsycInfo and Academic Search Index. The search was updated to November 7th, 2022.

The following search terms and their combinations were used: (“Corticobasal syndrome” OR “corticobasal degeneration” OR corticobasal OR cortico-basal OR CBS OR CBD) AND (“Cerebrospinal Fluid” OR cerebrospinal OR cerebro-spinal OR CSF OR liquor OR “fluid biomarkers” OR “serum biomarkers” OR “plasma biomarkers”). No limitations in the search strategy were applied to the publication date, study design, or language. References of considered studies were also explored to identify any further relevant data.

The records identified by the search were uploaded on “Rayyan” [18]. The titles and abstracts of the identified records were independently screened and selected by two authors (GR, EDS). Conflicts and disagreements were resolved by consensus.

The following set of predefined inclusion criteria was then individually applied to the selected full-text articles:

(i) recruiting patients with CBS,
(ii) analysed CSF values of biomarkers in CBS,
(iii) analysed plasma or serum biomarkers in CBS.

Preclinical studies, case reports, conference papers, abstracts, posters, letters, editorials, reviews and non-English papers were excluded.

A modified PRISMA Flow Diagram was used to report the flow process for study selection. Then, the Newcastle–Ottawa Scale (NOS) was applied to published trial studies for methodological and quality assessment [19]. Data extraction was performed by three reviewers (GR + EDS + AM).

The following information was abstracted from the retrieved papers: (1) demographic and clinical information (e.g., age, sex, disease duration, MMSE, UPDRS), (2) information about CSF and plasma biomarkers by dividing them into two groups: traditional or “novel” biomarkers. In our study we considered biomarkers as traditional when widely discussed in literature and used in clinical scenario, and “novel” when present only in a recent research context.

Descriptive statistic metrics extracted from the studies were used to report distributions of the parameters of interest. Data were reported as number (n), mean, standard deviations (SD), interquartile range (IQR) and 95% confidential interval (95% CI). Since biomarkers values were non-normally distributed in all selected studies, they adopted nonparametric Kruskal–Wallis test and pairwise Mann–Whitney test to assess fluid biomarkers differences between groups. Pearson coefficient index (r) and Kendall Tau coefficient (b) were used for correlation studies. Lastly, the ability of biomarkers to correctly categorize individuals into diagnostic groups was assessed using the receiver operator characteristic curves and corresponding area under the curve (AUC). The small sample size may be considered a limitation of included studies. Since CBS is a rare disease, the number of participants in the selected studies was tendentially small, decreasing the statistical power of the performed analyses. Therefore, the reported findings need to be interpreted with caution.

## Results

**Fig. 1:**
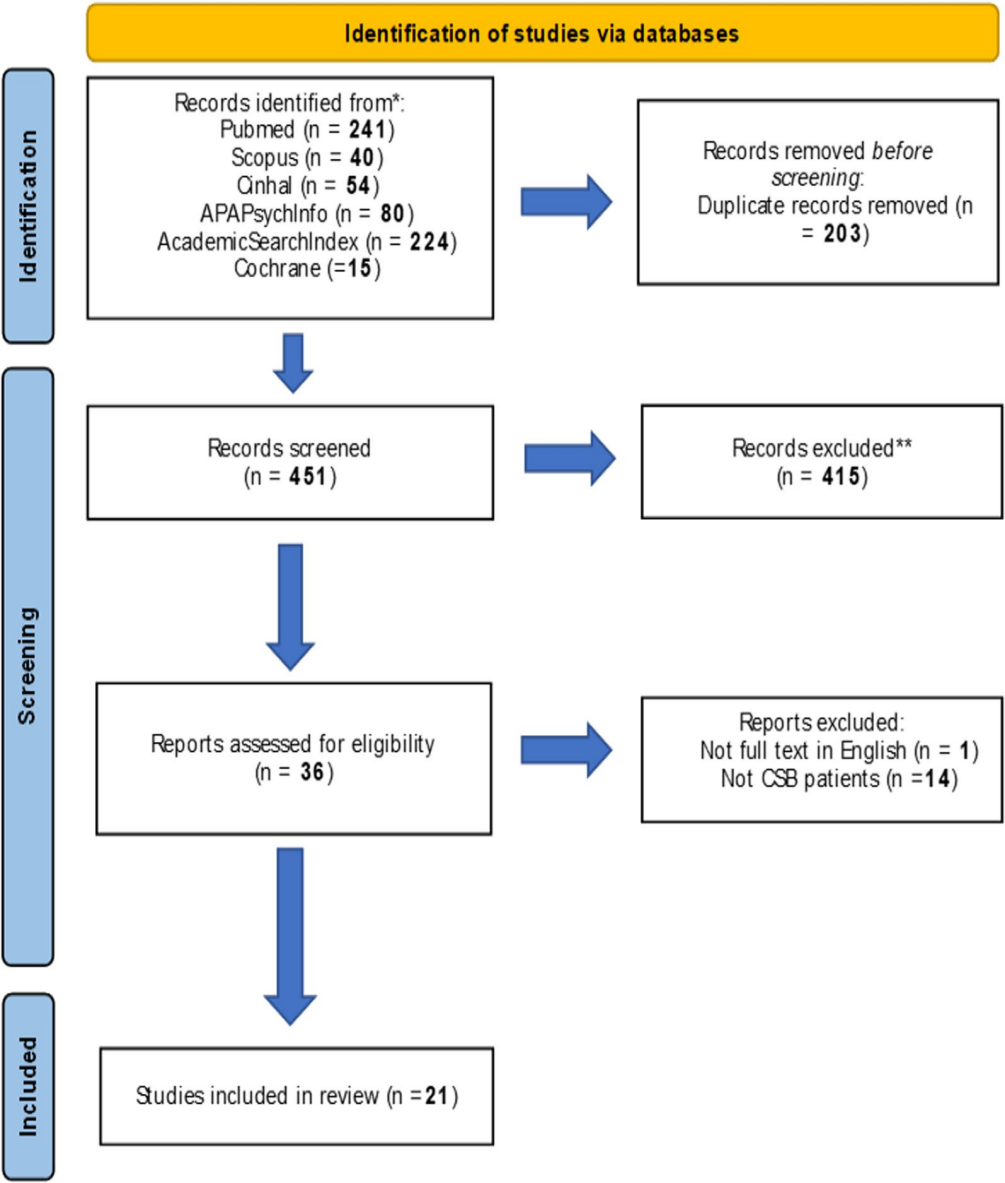
A PRISMA flow diagram of the study selection process

Bibliographic searches on literature databases yielded 654 records. After a first screening, 36 papers were selected. Of these, 15 were further excluded, as they did not meet the inclusion criteria. Overall, 21 studies were included (Fig. 1). All studies were retrospective studies, and no randomized clinical trials (RCTs) were identified.

A high consensus (> 90%) regarding the inclusion of the records was reported by the reviewers involved in the study selection process (GR + EDS) and conflicts in the screening process were resolved by consensus. The resume of the characteristics of the included studies is reported in Fig. 1.

### Quality assessment of the studies

The quality of the included studies was assessed through the Newcastle–Ottawa Scale (NOS) [19] and reported in Table 1. The assessment showed a high quality (7/9, 8/9) in all included studies. All the studies showed an adequate definition of cases and controls, satisfactory representativeness, and an adequate selection of controls. On the other hand, the principal reason associated with poorer quality was represented by comparability. Finally, high quality was also documented on exposure. In most studies, as reported in Table 2, CBS patients were selected according to Amstrong et al. criteria [8] and all studies evaluated small cohorts of patients: mean number 19 (*range 5–45*).

**Table 1:** Visual representation of the quality of the included studies assessed through the Newcastle–Ottawa Scale (NOS)

Study	Selection				Comparability			Exposure			Final score
	Case definition adequate	Representativeness of cases	Selection of controls	Definition of controls	Main factor	Additional factors		Ascertainment of exposure	Same method of ascertainment for cases and controls	Non response-rate	X out of 9
Olsonn et al. [31]	✓	✓	✓	✓				✓	✓	✓	7/9
Borroni et al. [37]	✓	✓	✓✓	✓		✓		✓	✓	✓	8/9
Magdalinou et al. 2015 [36]	✓	✓	✓	✓	✓	✓		✓	✓	✓	9/9
Hansson et al. [27]	✓	✓	✓	✓				✓	✓	✓	7/9
Hall et al. [28]	✓	✓	✓	✓				✓	✓	✓	7/9
Aerts et al. [15]	✓	✓	✓	✓	✓			✓	✓	✓	8/9
Benvenuto et al. [33]	✓	✓	✓	✓				✓	✓	✓	7/9
Meteer et al. [24]	✓	✓	✓	✓				✓	✓	✓	7/9
Alcolea et al. [29]	✓	✓	✓	✓				✓	✓	✓	7/9
Borroni et al. [26]	✓	✓	✓	✓		✓	✓	✓	✓	✓	8/9
Constantinides et al. [52]	✓	✓	✓	✓				✓	✓	✓	7/9
Luk et al. [35]	✓	✓	✓	✓		✓		✓	✓	✓	8/9
Boman et al. [22]	✓	✓	✓	✓	✓			✓	✓	✓	8/9
Jabbari et al. [34]	✓	✓	✓	✓				✓	✓	✓	7/9
Delaby et al. [25]	✓	✓	✓	✓				✓	✓	✓	7/9
Doss et al. [23]	✓	✓	✓	✓				✓	✓	✓	7/9
Quadalti et al. [20]	✓	✓	✓	✓		✓		✓	✓	✓	8/9
Bjorkhem et al. [32]	✓	✓	✓	✓				✓	✓	✓	7/9
Schulz et al. [30]	✓	✓	✓	✓		✓		✓	✓	✓	8/9

✓  = condition satisfied

**Table 2:** Demographic characteristics of the CBS patients evaluated in the included studies; diagnostic criteria by which CBS patients were selected and the number of patients evaluated in the studies are also enlisted

References	Subgroups	Diagnostic criteria	Total patients, No	Age, mean (SD)	Women (No)	Age of onset, mean (SD)	MMSE score, mean (SD)	UPDRS-III, mean (SD)	H & Y, mean (SD)	Disease duration, mean (SD)	Country
Olssonn et al. [31]		Amstrong et al	21	66 (7.6)	12	N	16.9 (2.4)	N	N	N	Sweden
Borroni et al. [37]		Toronto Criteria	16	61.3 (8.9)	6	58.9 (9.7)	25.2 (4.2)	15.9 (9.5)	N	1.73 (1.2)	Italy
Magdalinou et al. [36]		Cambridge Criteria	14	69.8 (N)	10	N	N	N	3.2 (N)	3.5 (N)	UK
Hansson et al. [27]	Lund cohort	Not defined	5	69 (4.9)	4	N	26.8 (2.9)	38.4 (26.7)	3.5 (1.7)	3.6 (1.3)	Sweden
	London cohort	Toronto Criteria	12	71 (7.2)	9	N	N	N	3.2 (1.0)	3.8 (2.2)	UK
Hall et al. [28]		Amstrong et al	6	67.8 (4.8)	4	N	28.3 (2.9)	8.8 (25.1)	3.3 (1.7)	3.3 (1.3)	Sweden
Aerts et al. [15]		Mayo Clinic Criteria	12	69 (N)	6	N	21.3 (6.8)	N	2.5 (N)	2 (N)	Netherlands
Benvenutto et al. [33]	CBS-A+	Amstrong et al	14	N	6	64 (N)	23 (N)	N	N	N	France
	CBS-A−		16	N	8	65.5 (N)	24 (N)	N	N	N	
Meeter et al. [24]		Amstrong et al	42	65 (N)	14	62 (N)	N	N	N	4 (N.)	Netherlands
Alcolea et al. [[29]		Amstrong et al	21	72.6 (6.9)	12	N	23.3 (6.7)	N	N	4.6 (2.5)	Spain
Borroni et al. [26]	CBS All	Amstrong et al	30	63.5 (8.9)	9	61 (9.3)	23.6 (6.1)	19.9 (11.5)	N	N	Italy
	CBS nAD-like		24	63.6 (9.8)	8	60.9 (10.3)	24.0 (6.2)	22.7 (10.7)	N	N	
	CBS AD-like		6	63.0 (4.0)	1	61.6 (4.3)	21.3 (5.8)	7.2 (4.4)	N	N	
	SPECT- nAD-like		18	62.2 (10.4)	7	60.6 (11.0)	25.1 (3.6)	21.6 (10.5)	N	N	
	SPECT- AD-like		5	63.6 (4.2)	0	62.6 (4.6)	23.0 (4.6)	6.7 (5.0)	N	N	
Constantinides et al. [52]		Amstrong et al	5	N	N	N	N	N	N	N	Greece
Luk et al. [35]	Cohort A	Not defined	5	73 (5.95)	N	N	N	N	N	N	Spain
	Cohort B		4	69.5 (8.8)	N	N	N	N	N	N	Germany
	Cohort C		8	72.6 (6.7)	N	N	N	N	N	N	Sweden
	Cohort D		5	59.23 (11.2)	N	N	N	N	N	N	Netherlands
	All cohorts		22	69.6 (8.6)	N	N	N	N	N	N	
	Cohort A + D		10	N. (N.)	N	N	N	N	N	N	
Boman et al. [22]	CBS + PSP patients	Amstrong et al	11	71 (N)	4	N	N	N	N	3 (N)	Sweden
Jabbari et al. [34]	CBS All	Amstrong et al	40	68.4 (7.4)	26	63.6 (7.2)	N	N	N	N	UK
	Unknown pathology		23	68.3 (6.8)	16	63.9 (7.6)	N	N	N	N	
	4RT		9	67.5 (8.6)	5	61.8 (9.6)	N	N	N	N	
	AD		8	69.7 (8.3)	5	64.6 (7.6)	N	N	N	N	
Delaby et al. [25]		Not defined	26	72 (7.3)	13	N	22.5 (5.3)	N	N	N	Spain
Doss et al. [23]	CBS + PSP patients	Not defined	11	N	N	N	N	N	N	N	Germany
Quadalti et al. [20]	CBS + PSP patients	Amstrong et al	58	71.2 (6.8)	27	N	25.1 (5.4)	38.9 (25.4)	2.7 (0.8)	N	Italy
Rodriguez et al. [52]	CBS + PSP patients	Amstrong et al	11	71.2 (N)	8	N	23.1 (6.7)	N	N	N	Spain
Bjorkhem et al. [32]		Amstrong et al	11	68.9 (5.4)	4	N	N	N	N	2.5 (1.7)	USA
Schulz et al. [30]		Amstrong et al	16	69.25 (5.6)	8	N	21.18 (6.10)	N	N	N	Germany
Di Stefano F et al. [51]	CBS	Modified Cambridge Criteria	45	69.2 (7.5)	22	65.9 (7.4)	21.5 (6.5)	N	N	3.2 (1.8)	France
	CBS AD +		8	66.25 (7.8)	3	N	21.25 (4.5)	N	N	3.87 (2.29)	
	CBS AD -		37	65.83 (7.4)	19	N	21.52 (6.8)	N	N	3.16 (1.7)	

*No* number of patients, *SD* standard deviation, *UPDRS-III* Unified Parkinson Disease Rating Scale—III version, *H & Y* Hoehn and Yahr Scale

*N* data not available, *CBS* corticobasal syndrome, *CBS-A+* CBS with underlying amyloid pathology, *CBS-A−* CBS not associated with amyloid pathology, *CBS AD-like* CBS with fluid biomarkers profile suggestive of AD pathology, *CBS nAD-like* CBS with fluid biomarkers profile not suggestive of AD pathology, *SPECT AD-like* CBS with Single Photon Emission Computed Tomography (SPECT) profile suggestive of AD pathology, *SPECT nAD-like* CBS with Single Photon Emission Computed Tomography (SPECT) profile not suggestive of AD pathology

### Demographics characteristics

Of 315 patients with available information on sex, 54% (*173*) were women. Mean age of CBS cohorts, where reported, was 68.4 years (*range 61.3–72.6*), with a mean age at onset of 63 years (*range 58.9–65.9*) and a mean disease duration of 3.2 years (*range 1.73–4.6*). Where reported, mean MMSE, UPDRS-III, and H&Y scores resulted in 23.13 (*range 16.9–28.3*), 20.75 (*range 8.8–38.4*), and 3.2 (*range 1.73–4.6*) respectively. The above data does not consider studies where mixed cohorts of CBS/PSP patients were evaluated [20–23]. Almost all studies were conducted in Europe, with only one study performed in the US.

### Value of traditional CSF biomarkers in CBS patients: Ab42, T-tau and P-tau

Findings on traditional CSF biomarkers, including NfL, are reported in Table 3.

**Table 3:** Studies, in which classical cerebrospinal fluid (CSF) biomarkers were evaluated, are enlisted; if reported, values of Beta-Amyloid 42 (Ab42), Alpha-Synuclein (a-syn), Total-Tau (T-Tau), Phospho-Tau (P-Tau), Neurofilament Light Chains (NfL) and the detection method are described

References	Subgroups	CSF Ab42 pg/ml mean (*)	CSF a-syn pg/ml mean (*)	CSF T-Tau pg/ml mean (*)	CSF P-Tau pg/ml mean (*)	CSF NfL pg/ml mean (*)	Methods
Olssonn et al. [31]		255 [166–293]	N	71 [62–108]	21 [16–28]	1281 [828–2713]	Luminex: Ab42 + T-Tau + P-Tau ELISA: NfL
Magdalinou et al. [36]		715 (553–878)	1497 (1183–1811)	286 [234–381]	38 [30–45]	1937 [1465–3434]	Fujirebio: Ab42 + T-Tau + P-Tau ELISA: NfL + a-syn
Hansson et al. [27]	Lund cohort	538 (288)	N	358 (93)	58.3 (18.4)	2.498 (848)	Fujirebio: Ab42 + T-Tau + P-Tau ELISA: CSF NfL
	London cohort	745 (295)	N	425 (289)	47.3 (21)	2.845 (2.269)	
Hall et al. [28]		380 [234.5–611.1]	N	420.8 [219.8–661.4]	39.9 [24.8–54.8]	N	ELISA: Ab42 + T-Tau + P-Tau
Aerts et al. [15]		730 (316)	N	402 (199)	48 [38–59]	N	ELISA: Ab42 + T-Tau + P-Tau
Benvenutto et al. [33]	CBS-A +	361 [307–397]	N	593 [348–809]	83 [65–123]	N	ELISA: Ab42 + T-Tau + P-Tau
	CBS-A-	843 [521.25–1140.75]	N	279.5 [193.75–3479.25]	45.5 [33.5–61.5]	N	
Meeter et al. [24]		810 [607–999]	N	336 [246–446]	47 [38–57]	2664 [1715–4158]	Fujirebio: Ab42 + T-Tau + P-Tau ELISA: NfL
Alcolea et al. [29]		480.1 (165.3)	N	279.5 (108)	43.4 (13.3)	2,264.3 (1216.5)	ELISA: Ab42 + T-Tau + P-Tau + NfL
Borroni et al. [26]	CBS	748.2 (431.5)	N	409.1 (285.3)	N	N	ELISA: Ab42 + T-Tau
	CBS nAD-like	865.2 (402.5)	N	337.7 (242.5)	N	N	
	CBS AD-like	280.2 (77.3)	N	694.7 (281.7)	N	N	
	SPECT- nAD-like	888.9 (412.5)	N	308.1 (223.1)	N	N	
	SPECT- AD-like	266.1 (77.2)	N	726.5 (302.7)	N	N	
Boman et al. [22]	CBS + PSP patients	N	N	N	N	N	Western blot
Delaby et al. [25]		696 [479–911]	N	302 [209–424]	51 [40–64]	1637 [923–2797]	Fujirebio: Ab42 + T-Tau + P-Tau ELISA: NfL
Quadalti et al. [20]	CBS + PSP patients	655 [476.3–877.0]	N	195 [157.3–282.3]	29 [22.3–37.8]	1569 [1120–2128]	Fujirebio: Ab42 + T-Tau + P-Tau Quanterix: NfL
Rodriguez et al. [52]	CBS + PSP patients	524.6 (193.4)	N	244.8 (112.6)	41.2 (15)	N	Fujirebio: Ab42 + T-Tau + P-Tau
Schulz et al. [30]		N	714.87 (179.91)	70.58 (39.06)	N	4595.31 (3635.94)	ELISA: a-syn Quanterix: T-Tau + P-Tau + NfL
Di Stefano F et al. [51]	CBS	N	N	N	N	N	Fujirebio: Ab42 + T-Tau + P-Tau
	CBS AD +	288 (97)	N	655 (324)	113 (63)	N	
	CBS AD -	573 (212)	N	317 (157)	46 (19)	N	

All biomarkers enlisted were evaluated in cerebrospinal fluid (CSF)

(*) data reported as: (x) = standard deviation (SD); [x–y] = Interquartile Range (IQR); (x–y) = 95% Confidence Interval (95% CI)

*N* data not available, *CBS-A+* CBS with underlying amyloid pathology, *CBS-A−* CBS not associated with amyloid pathology, *CBS AD-like* CBS with fluid biomarkers profile suggestive of AD pathology, *CBS nAD-like* CBS with fluid biomarkers profile not suggestive of AD pathology, *SPECT AD-like* CBS with Single Photon Emission Computed Tomography (SPECT) profile suggestive of AD pathology, *SPECT nAD-like* CBS with Single Photon Emission Computed Tomography (SPECT) profile not suggestive of AD pathology

CSF Ab42 levels resulted lower in CBS [*patients number (n)* = *42 and 26*] when compared with healthy controls (*n* = *92 and 108 respectively*) (*p* < *0.001*) [24, 25]; although in one study, Ab42 levels of 32 CBS patients resulted within normal range, due to a significant representation of CBD (n = 26) rather than CBS-AD (n = 4) patients [26]. In 12 CBS patients CSF Ab42 levels showed an inverse correlation with MMSE (*r* = *0.481, p* < *0.05*) [15]; also blood NfL levels showed a correlation with MMSE in 2 distinct cohorts (*b* = − *2.01, p* = *0.001; b* = − *0.182, p* = *0.034*) composed by, respectively, 12 and 5 patients [27].

CSF T-Tau and P-Tau levels resulted increase in CBS (*n* = *12 and 16*) patients compared to healthy controls (*n* = *49 and 108; p* < *0.001*) [15, 25]. In other studies, 12 CBS patients showed higher levels of T-Tau and P-Tau compared to 21 PSP, and 28 PD patients [15], 42 CBS patients showed higher levels of T-Tau and P-Tau compared to 64 PSP patients, but the difference was not significant [24]. T-Tau levels also resulted higher in CBS (*n* = *6*) than in DLB (*n* = *11*; *T-Tau: p* < *0.001; P-Tau: p* < *0.05)* [28]. On the other hand, CSF T-Tau and P-Tau levels resulted reduced when comparing 21 CBS patients to 72 AD patients (*p* < *0.001*) [29].

Schultz et al. attributed a good accuracy to T-Tau when differentiating 16 CBS patients versus 20 healthy controls, 151 PD, and 38 PSP patients (*AUC 0.722, 0.722, 0.741 respectively*) [30]. A linear but weak correlation was also described in CBS (*n* = *17*) between CSF T-Tau and blood NfL (*b* = *0.151, p* = *0.020*) [27], although not confirmed in another study in 16 CBS patients [31]. In a mixed cohort of 11 CBS and PSP patients, T-Tau levels showed a correlation with progranulin (*r* = *0.192, p* = *0.001*) [21]; furthermore, in a cohort of 11 CBS patients, T-Tau levels correlated with 24- S-Hydroxycholesterol, 24- OHC (*r* = *0.98, p* < *0.001),* whilst no significant association was found with 27- S-Hydroxycholesterol (27-OHC) levels, suggesting a direct interaction between the neuronal production of 24-OHC and T-Tau [32].

Previous studies also described a linear correlation between P-Tau and progranulin levels *(r* = *0.201, p* < *0.001* [21], P-Thr181Tau and 24-OHC *(r* = *0.98, p* < *0.001),* thus furtherly supporting the above-mentioned hypothesis [32]. An inverse correlation with MMSE was also reported for P-Tau *(r* = − *0.642, p* = *0.001)* in 12 CBS patients [15].

### Value of NfL in CBS patients

CSF NfL levels resulted higher in CBS (n = 26) when compared to healthy controls (*n* = *118; p* < *0.01)* [25], and in CBS (*n* = *21*) compared with PD (*n* = *29, p* < *0.01*), PD-MCI (*n* = *19, p* < *0.01*) [31], but lower when comparing CBS (*n* = *26*) to ALS (*n* = *68, p* < *0.01*) [25]. Furthermore, NfL levels in 16 CBS non-patients resulted higher than in 14 CBS-AD patients (*p* < *0.01*) [33].

According to Hansson et al. [27], CSF NfL resulted highly discriminative of 171 PD from 5 CBS patients (AUC 0.96). Quadalti et al. [20] proposed a CSF NfL cut-off of 1057 pg/ml for differentiating 116 PD from 58 CBS/PSP patients with a sensitivity of 97.4% and a specificity of 80.8% (*p* < *0.001*). Accordingly, Schulz et al. [30] showed that CSF NfL levels discriminate with good accuracy 16 CBS patients from 38 PSP (AUC 0.93) and 45 DLB (AUC 0.806) patients.

Several studies documented a strong correlation between CSF and blood NfL in CBS [20, 27, 30]. Blood NfL levels resulted higher in a mixed cohort of 58 CBS and PSP patients and a cohort of 12 CBS patients when compared to, respectively, 72 and 26 healthy controls (*p* < *0.001*), 116 and 20 PD patients (*p* < *0.001*) [20, 27]; furthermore, blood NfL levels resulted higher in 40 CBS patients than in 101 PSP patients (*p* < *0.001*) [34]. As reported by Hansson et al. [27] blood NfL differentiated PD (n = 20) and healthy controls (*n* = *26*) from CBS *(n* = *12)* with a sensitivity of 82% and a specificity of 92% (*AUC 0.92; 95%CI 0.88–0.95*) in one cohort, and PD *(n* = *171)* and healthy controls *(n* = *53)* from CBS *(n* = *5)* with a sensitivity of 93% and a specificity of 83% (*AUC 0.9; 95% CI 0.85–0.98*) in a second cohort. A blood NfL cut-off of 16.6 pg/ml allowed to discriminate 20 PD patients from 58 CBS/PSP patients with a sensitivity of 88.7% and a specificity of 87.8% (*AUC 0.936*) [20].

### Value of emerging CSF and blood biomarkers in CBS patients

Finding on emerging CSF and blood biomarkers, including blood NfL, are enlisted in Table 4.

**Table 4:** Studies, in which emerging cerebrospinal fluid (CSF) and blood biomarkers were evaluated, are enlisted. The detection methods of the different biomarkers are also described

References	Subgroups	Other biomarkers, mean (*)	Methods
Borroni et al. [37]		CSF **Tau Form Ratio (33 kDa/55 kDa)** 0.997 (0.34); (0.815–1.180)	Western Blot
Magdalinou et al. [36]		CSF **sAPPb** 238 ng/ml (167–309) CSF **YKL-40** 21.5 ng/L × 10^4 (17.3–25.8) CSF **MCP-1** 531 ng/L (406–655) CSF **sAPPa** 394 ng/ml (217–516)	MesoScale Discovery: sAPPb + MCP-1 + sAPPa ELISA: YKL-40
Hall et al. [28]		CSF **Neurogranin-EL** 370.2 pg/ml [167.4–525.2] CSF **Neurogranin-UGOT** 174 pg/ml [107.5- 285] CSF **BACE-1** 1693 pg/ml [1052–2722.4] CSF **NfH** 0.799 pg/ml [0.649–1.083]	ELISA: Nerugranin-El + Neurogranin-UGOT + BACE-1 + NfH
Aerts et al. [29]		CSF **Lactate** 1666 micromol/L [1437–1808] CSF **Total Proteins** 488 mg/L (126) CSF **Ab42/T-Tau** 2.28 [0.64–3.69] CSF **Ab42/P-Tau** 12.9 (7.1) CSF **P-Tau/T-Tau** 0.18 [0.13–0.2]	ELISA: Ab42 + T-Tau + P-Tau
Benvenutto et al. [33]	CBS-A+	CSF **Ab42/Ab40** 0.06 [0.04–0.07] CSF **P-Tau/Ab42** 0.26 [0.19–0.38] CSF **T-Tau/Ab42** 1.7 [1.16–2.49] CSF **Ab42/P-Tau** 3.89 [2.67–5.21]	ELISA: Ab42 + Ab40 + T-Tau + P-Tau
	CBS-A−	CSF **Ab42/Ab40** 0.14 [0.10–0.16] CSF **P-Tau/Ab42** 0.05 [0.04–0.07] CSF **T-Tau/Ab42** 0.33 [0.22–0.51] CSF **Ab42/P-Tau** 21.12 [14.62–26.68]	
Meeter et al. [24]		CSF **P-Tau/T-Tau ratio** 0.13 [0.11–0.16]	Fujirebio: P-Tau + T-Tau
Alcolea et al. [29]		CSF **sAPPb** 556.4 ng/ml (226.9) CSF **YKL-40** 280.6 ng/ml (60.4) CSF **sAPPb/YKL-40** 2.0 (0.8) CSF **NfL/sAPPb** 4.3 (2.6)	ELISA: sAPPb + YKL-40 + NfL
Borroni et al. [26]	CBS	CSF **T-Tau/Ab42** 0.86 (1.03)	Fujirebio: T-Tau + Ab42
	CBS nAD-like	CSF **T-Tau/Ab42** 0.43 (0.29)	
	CBS AD-like	CSF **T-Tau/Ab42** 2.60 (1.20)	
	SPECT- nAD-like	CSF **T-Tau/Ab42** 0.44 (0.46)	
	SPECT- AD-like	CSF **T-Tau/Ab42** 2.83 (1.10)	
Luk et al. [35]	Cohort A	CSF **3R-Tau** 25 pg/ml "5–27" CSF **4R-Tau** 30 pg/ml "20–60"	RD3 and RD4 monoclonal antibodies + immuno-PCR: 3R-Tau, 4R-Tau
	Cohort B	CSF **3R-Tau** 2 pg/ml "1–3" CSF **4R-Tau** 10 pg/ml "5–15"	
	Cohort C	CSF **3R-Tau** 25 pg/ml "10–50" CSF **4R-Tau** 5 pg/ml "4–8"	
	Cohort D	CSF **3R-Tau** 5 pg/ml "4–10" CSF **4R-Tau** 0 pg/ml "0"	
	All cohorts	CSF **3R-Tau** 20 pg/ml "0–50" CSF **4R-Tau** 10 pg/ml "20–60"	
	Cohort A + D	CSF **4RTau/T-Tau** 0.037 (0.011) CSF **4R-Tau/P-Tau** 0.168 (0,092)	
Doss et al. [23]	CBS + PSP patients	CSF **NMDAR Ab** (IgA, IgM or IgG) **positivity percentage** 54.5% CSF **NMDAR mean titre** 1:10	Recombinant Immunofluorescence Assays
Quadalti et al. [20]	CBS + PSP patients	blood **NfL** 26.6 pg/ml [19.4–40.8] CSF **Ab40** 8304 pg/ml [5761–10664] CSF **Ab42/Ab40** 0.86 [0.77–0.94] **a-syn RT-QuIC**: no seeding activity	Quanterix: blood NfL Fujirebio: Ab40 + Ab42 RT-QuIC: a-syn seeding activity
Rodriguez et al. [52]	CBS + PSP patients	CSF **YKL-40** 273.8 ng/ml (57.5) CSF **Progranulin** 5.2 ng/ml (1.3)	Fujirebio: YKL-40 ELISA: Progranulin
Schulz et al. [30]		blood **aSyn** 6548.94 pg/ml (2623.41) CSF **pS129 aSyn** 2.07 pg/ml (0.83) blood **NfL** 51.59 pg/ml (33.80) CSF **NfH** 1.14 ng/ml (0.82) CSF **UCHL-1** 2063.21 pg/ml (517.12) CSF **GFAP** 19687.24 pg/ml (5320.02) blood **GFAP** 290.42 pg/ml (165.23) CSF **S100B** 3.78 pg/ml (1.08) blood **S100B** 0.09 pg/ml ( 0.05) CSF **sTREM2** 7170.38 pg/ml (3313.50) blood **sTREM2** 5837.98 pg/ml (4072.41) CSF **YKL-40** 177,413.75 pg/ml (64,746) blood **YKL-40** 46,912.94 pg/ml (23,763)	ELISA: aSyn + blood aSyn + NfH ELISA: UCHL-1 + GFAP + blood GFAP ELISA: S100 B + sTREM2 + YKL-40 + blood YKL-40 Quanterix: blood NfL
Di Stefano et al. [51]	CBS	N	Fujirebio: Ab42 + T-Tau + P-Tau
	CBS AD+	CSF **T-Tau/Ab42** 2.49 (1.33) CSF **P-Tau181/Ab42** 0.43 (0.25)	
	CBS AD−	CSF **T-Tau/Ab42** 0.59 (0.3) CSF **P-Tau181/Ab42** 0.09 (0.04)	

*CSF* biomarkers were detected in cerebrospinal fluid, *blood* biomarkers were detected

(*) data reported as: (x) = standard deviation (SD); [x–y] = Interquartile Range (IQR); (x–y) = 95% Confidence Interval (95% CI)

*CBS-A+* CBS with underlying amyloid pathology, *CBS-A−* CBS not associated with amyloid pathology

*sAPPb* soluble Amyloid Precursor Protein b, *YKL-40* Mammalian Chitinase-Like Protein-40, *MCP-1* Monocyte Chemoattractant Protein-1, *sAPPa* soluble Amyloid Precursor Protein a, *BACE-1* Beta-Secretase-1, *NfH* Neurofilament Heavy chains, *3R Tau *3 Repeats Tau, *4R Tau* 4 Repeats Tau, *NMDAR Ab* Antibodies Anti-NMDA Receptors, *RT-QuIC* Real-Time Quacking Induced Conversion, *UCHL-1* ubiquitin C-terminal hydrolase L1, *GFAP* Glial Fibrillary Acid Protein, *S100B* S100 calcium-binding protein B, *sTREM2* triggering receptor expressed on myeloid cells 2, *Ab42* Beta-Amyloid 42, *T-Tau* Total-Tau, *P-Tau* Phospho-Tau, *NfL* Neurofilament Light Chains, *aSyn* alpha-Synuclein

In terms of additional biomarkers, Hall et al. [28] showed decreased CSF levels of neurogranin in all atypical parkinsonism (APS), except for CBS (*p* < *0.05*) and DLB, compared to healthy controls and AD. Pairwise comparisons showed significantly higher levels of 24-OHC in 11 CBS patients compared with 19 controls (*p* < *0.01*) [32], while a significant difference in 4R-Tau CSF levels between controls, CBS, PSP, and AD was not identified [35].

According to Luk et al. [35], 4R-Tau mean levels resulted lower in CBS (*n* = *8 and 5*) than in healthy controls (*n* = *12 and 9*), but higher in CBS than in PSP (*n* = *9 and 12*) and AD (*n* = *11*) in two cohorts. Two further cohorts confirmed the trends, but no statistically significant differences were observed. Mean 3R-Tau CSF levels were also evaluated, and no differences were observed between different diagnostic groups. Therefore, Tau isoforms in the brain may not be reflected in CSF levels.

According to Schulz et al. [30], five biomarkers showed an elevated accuracy in differentiating 16 CBS patients from 38 PSP patients and 20 healthy controls, namely CSF NfH (*AUC 0.9*), S100B (*AUC 0.9*), CSF ubiquitin C-terminal hydrolase-1 UCHL-1 (*AUC 0.84*), CSF Glial Fibrillary Acid Protein (GFAP) (*AUC 0.8*), CSF soluble triggering receptor expressed on myeloid cells (sTREM2) (*AUC 0.96*), and serum S100 calcium-binding protein B (S100B) (*AUC 0.84*). Furthermore, CSF Neurofilament Heavy Chains (NfH) showed a good accuracy in differentiating CBS from 45 DLB patients (*AUC 0.9*), and CSF S100B in differentiating CBS from 17 MSA patients (0.8).

The combination of 9 different CSF biomarkers [36] [namely soluble amyloid precursor protein a (sAPPa), soluble amyloid precursor protein b (sAPPb), Mammalian Chitinase-Like Protein-40 (YKL), Monocyte Chemoattractant Protein-1 (MCP-1), NfL, P-Tau, T-Tau, a-synuclein (a-syn), and Ab42] differentiates with a good accuracy 14 CBS patients from 31 PD patients (*AUC 0.98, 95%CI 0.97–0.99*), from 26 AD and 16 FTD patients (*AUC 0.93, 95%CI 0.85–0.99*).

The ratio between the 33KDa and the extended 55 KDa truncated Tau forms (Tau Ratio, 33 KDa/55 KDa) resulted lower in 18 PSP than 16 CBS patients with excellent accuracy (*AUC 0.91*) in differentiating these disorders [37]. A significant difference was found in Ab42/T-Tau ratio comparing 12 CBS and 21 PSP patients *(0.86, 95% CI 0.74–0.98)* [15] In addition, Lysosomal network proteins LC3, EEA1 and lysozyme levels resulted higher in 10 CBS patients compared to healthy controls [22]; however, the difference resulted statistically significant only for LC3 (*92% higher in CBS than controls; p* < *0.001*). Antibodies Anti-NMDA Receptors (NMDAR) (IgA, IgM, IgG) percentage and mean titre were evaluated in several neurodegenerative disorders, including CBS [23].

Finally, CSF a-syn levels were evaluated in two studies [30, 36], in which they showed a low sensitivity and specificity in differentiating CBS from AD, PD, other APS, ALS, and healthy controls. Shultz et al. [30] found no significant correlation between CSF and serum a-syn levels; similarly, to CSF a-syn, serum a-syn levels did not help to discriminate CBS patients from healthy controls and patients with a diagnosis of other neurodegenerative diseases. In a mixed group of 58 CBS/PSP patients, Real-Time Quaking Induced Conversion (RT-QuIC) analyses documented no seeding activity for a-syn and the combination of a-syn RT-QuIC with CSF NfL levels discriminated 116 PD patients from PSP/CBS with an accuracy of 99% (*p* < *0.01*).

## Discussion

CBS is a rare neurodegenerative disorder presenting a progressive, asymmetrical, akinetic rigid syndrome and early cortical signs. However, its clinical and pathological heterogeneity combined with its rarity and the lack of substantial autopsy studies complicate the understanding of this syndrome.

Among the several pathological substrates underlying CBS, CBD accounts for less than half of CBS antemortem diagnosis, and PSP and AD come as close second and third most common cause, respectively [2]. An example of the non-specificity of the CBS clinical phenotype in predicting the definitive neuropathological diagnosis is the report by Koga et al.: within 21 cases with clinical CBS diagnosis, only 5 had pathologically confirmed CBD [2]. Indeed, the sensitivity of clinical findings for predicting underlying CBS pathology ranges from 26.3 to 56%: patients with CBS-AD were averagely younger than CBS-CBD at onset, myoclonus and tremor were more frequent in CBS-AD and CBS-CBD, respectively [38]. However, these findings were not widely replicated in other studies and other elements did not allow an “in vivo” differentiation of these conditions (e. i: family history tends to be negative in CBS presentations of AD and CBS-CBD).

Imaging findings help in the diagnosis of CBS. Benvenutto et al. [33] described two different phenotypes of CBS: one ‘parietal’ or posterior profile associated with the presence of amyloid biomarkers, and the other ‘pre-motor’ or anterior profile without amyloidosis. This finding is consistent with the hypothesis of an anteroposterior gradient of CBD [39, 40]. Interestingly, both CBS-CBD and CBS-AD demonstrated relative sparing of the hippocampal cortex, which may be expected in CBD but is atypical in AD pathology, raising several diagnostic doubts [41].

Amyloid-PET positivity, while being a candidate for the diagnosis of CBS-AD, might not reflect an exclusive AD neuropathological substratum, owing to possibly overlapping neurodegenerative conditions (i.e., AD-CBD, AD-PSP); additionally, false positives can occur due to age-related amyloid deposition that may occur in some healthy elderly patients. Recently, Tau protein also became a target for in vivo molecular diagnosis. Several Tau PET tracers have been developed. Tau PET may help distinguish tauopathy-CBS from non-tauopathy-CBS, and AD-CBS from non-AD tauopathies; however, it remains challenging to differentiate between non-AD tauopathies, particularly CBD and PSP [2].

Against this background, identifying fluid biomarkers to rely upon for the in vivo pathological diagnosis becomes paramount.

As expected, in our results, Ab42 is reduced in CBS-AD cases versus CBS non-AD-like, and CBS-AD seems to be associated with a more severe amyloid pathology than the classical AD-pattern. Whereas low Aβ42 has a high predictive value for AD pathology [42], CSF biomarkers may be helpful as amyloid-labelled imaging in predicting AD in patients presenting with CBS.

According to our findings, because a-synucleinopathy is not involved in the pathogenesis of CBS, the evidence of a-synucleopathy should question a diagnosis of CBS [30].

Considering CBS populations independently from hypothetical pathogenesis, T-Tau and P-Tau levels were higher than PSP, PD and DLB, and inferior to AD. Elevated Tau levels in CBS may be attributed to a high percentage of CBS-AD patients, with non-AD-like CBS being the reason for lower levels compared to AD. An interesting hint emerges from Bjorkhem et al. [32]: they found a strong correlation between T-Tau, P-Tau and 24-OHC levels, a sidechain oxidised metabolite of cholesterol, which is released in CSF by necrotising cells, suggesting a direct interaction in the neuronal production of the three factors, likely owing to abnormal CYP46A1 activity. Additionally, the correlation was more evident in CBS than in PD, suggesting more severe neurodegeneration in CBS patients.

Longitudinal analysis in Alzheimer Disease Neuroimaging Initiative (ADNI) [43] showed a significant association between Tau levels and worse cognition, greater atrophy and lower hypometabolism during follow-up. While elevated P-Tau levels seem to be restricted to AD pathology, T-Tau levels are altered in a disease-specific pattern in several neurodegenerative conditions, including non-primary tauopathies. In all these conditions T-Tau levels have shown a significant prognostic value [44].

NfL levels in CBS were higher than in other neurodegenerative diseases, except for ALS [31]. The absence of correlation with MMSE seemingly suggests that NfL elevation in CSF is an epiphenomenon of neurodegenerative events related to motor impairment rather than cognitive decline, as suggested by the correlations evidenced in CBS patients between blood NfL and UPDRS and H&Y scores. Moreover, higher levels of CSF NfL were associated with poorer survival (*p* = *0.001*) [24]. Therefore, NfL might be a candidate biomarker for detecting a neurodegenerative process presenting with a CBS phenotype.

Jabbari et al. [34] evidenced significantly higher levels of NfL in CBS cases with confirmed 4RT pathology when compared to CBS cases with AD pathology, suggesting NfL’s potential clinical usefulness in discriminating CBS-AD and CBD.

The potential usefulness of NfL levels in predicting CBS severity and progression may be derived from evidence regarding other neurodegenerative dementias[41]; indeed, high CSF and plasma NfL levels have been associated with MCI secondary to AD [45, 46] and with more severe disease in AD patients [47]. Blood NfL were also correlated with blood and CSF T-Tau and P-Tau levels in AD patients [43, 48].

Given the widespread axonal degeneration characteristic of APS, CSF and blood NfL may indirectly gauge the effectiveness of treatment, especially in CBS where a marked elevation of CSF and blood levels has been observed. NfL has already served as an endpoint in clinical trials; for example, in multiple sclerosis, a dynamical decrease of CSF NfL was observed in clinical trials with Fingolimod [49] and a reduction of blood NfL has been documented in clinical trials with Tofersen in ALS patients [50]. Thus, NfL may become a surrogate endpoint for future therapeutic trials in CBS.

Budding evidence for other CBS biomarkers was provided by the study by Schulz et al. [30] where CBS and AD could be differentiated through fluid biomarkers solely via a combination of nine different proteins: sAPPb, sAPPa, YKL, MCP-1, NfL, P-Tau, T-Tau, a-syn, and Ab42. Furthermore, Magdalinou et al. [36] results suggested that APPa metabolism is altered differently in CBS and AD. Unexpectedly, despite the 4RT alteration of CBS-CBD and the mixed 4RT-3RT accumulation in AD, no significant increase of 4RT and 3RT CSF levels was found in CBS patients compared to the controls. Given this, Tau isoforms accumulating in the brain might not reflect CSF levels. Therefore, gauging neuropathologically specific Tau isoforms might be fruitless in clinical practice as they do not discriminate the underlying pathogenesis of CBS patients.

No study considering progranulin as a neuropathological biomarker was found, despite CBS may be a manifestation of the GRN-FTD spectrum associated with an underlying TDP43 pathology. Accordingly, genetic analysis would be appropriate to categorize certain types of patients, especially those between the ages of 50 to 60. In such cases of CBS an early involvement of frontal and temporal cortex is to be expected [4]. Therefore, neuropsychological testing may demonstrate early impairment in frontal lobe tasks or specific language dysfunction before the onset of frank dementia. Additionally, behavioural disturbances represent a common early feature.

In conclusion, integrating fluid biomarkers, genetic testing, and imaging findings may enhance diagnostic accuracy in routine clinical practice by identifying in vivo the underlying pathological processes, and the neuronal functions more likely to be impaired during disease evolution. Biomarkers might provide clinical benefits in terms of an earlier access to classical and potential disease-modifying therapies (i.e., it may be reasonable to avoid potential amyloid-targeted therapies in the absence of amyloid pathology).

Given their informative capacity, future trial designs should consider baseline CSF NfL dosage to stratify treatment arms according to neuropathological substrates, and serum NfL dosage might be used to monitor the evolution of CBS. In this sense, more prospective cohort studies are needed to define the reference cut-offs. Within these biomarkers-centred trials, however, neuropsychological tests (i.e., quantification of apraxia and aphasia, assessment of eye movements) would remain essential, given the possible co-morbidity between CBS patient groups and the common occurrence of amyloid pathology in ageing populations as well as other pathological conditions (i.e., DLB). In this sense, it would also be helpful to define standardized clinical criteria to define CBS; however, we do not consider that the current heterogeneity of criteria may have influenced our results since Armstrong's criteria were adopted in almost all assessed studies. Even if 3 studies adopted different criteria, respectively Mayo Clinic, University of Toronto and Modified Cambridge Criteria, all these share with Amstrong's paradigm the same categorisation of clinical motor features defining CBS. However, cognitive and language impairment were accounted differently.

Despite a systematic approach, our review and the studies included bear their own biases. Most studies investigated very heterogeneous and limited cohorts of patients: only 3 studies, were limited to CBS patients, and a limited proportion of studies reported a post-mortem autoptic confirmation of any possible or probable neurodegenerative disorder according to clinical and biological features [26, 33, 34]. Four studies evaluated cohorts including CBS and PSP patients [20–23]. Additionally, AD-confirmed and non-AD CBSs were not clearly differentiated within the studies. The relative distributions and correlations of biomarkers levels are hard to define in such groups. All included studies are retrospective, and direct causative relations cannot be determined. Further prospective studies are necessary to collect reliable evidence to characterise the CBS clinical phenotype.

### Conclusions and future perspectives

CBS is a clinical syndrome caused by various underlying disorders, with the most recurring pathologies at autopsy being CBD and PSP.

In the future, it may be possible to deduce the underlying neurodegenerative disorder during life (without anatomopathological confirmation) through a combination of clinical symptoms, signs, and neuropsychological tests, with supporting information from various biochemical and imaging biomarkers.

For this purpose, it is necessary to conduct prospective cohort studies, starting with neurological examination and neuropsychological tests, to assess the correlations of clinical profiles and various biomarkers. This integration into disease classification may be the key to an accurate diagnosis and appropriate patient selection for future clinical trials.

## Data Availability

Not applicable.

## References

[1] Parmera JB, Rodriguez RD, Neto AS, Nitrini R, Brucki SMD (2016). Corticobasal syndrome: A diagnostic conundrum. Dementia & neuropsychologia.

[2] Koga S, Josephs KA, Aiba I, Yoshida M, Dickson DW (2022). Neuropathology and emerging biomarkers in corticobasal syndrome. Journal of Neurology, Neurosurgery and Psychiatry.

[3] Kouri N, Whitwell JL, Josephs KA, Rademakers R, Dickson DW (2011). Corticobasal degeneration: A pathologically distinct 4R tauopathy. Nature Reviews Neurology.

[4] Baker M, Mackenzie IR, Pickering-Brown SM, Gass J, Rademakers R, Lindholm C, Snowden J, Adamson J, Sadovnick AD, Rollinson S, Cannon A (2006). Mutations in progranulin cause tau-negative frontotemporal dementia linked to chromosome 17. Nature.

[5] Benussi L, Binetti G, Sina E, Gigola L, Bettecken T, Meitinger T, Ghidoni R (2008). A novel deletion in progranulin gene is associated with FTDP-17 and CBS. Neurobiology of Aging.

[6] Le Ber I, Camuzat A, Hannequin D, Pasquier F, Guedj E, Rovelet-Lecrux A, Hahn-Barma V, van Der Zee J, Clot F, Bakchine S, Puel M (2008). Phenotype variability in progranulin mutation carriers: A clinical, neuropsychological, imaging and genetic study. Brain.

[7] Arienti F, Lazzeri G, Vizziello M, Monfrini E, Bresolin N, Saetti MC, Picillo M, Franco G, Di Fonzo A (2021). Unravelling genetic factors underlying corticobasal syndrome: A systematic review. Cells.

[8] Armstrong MJ, Litvan I, Lang AE, Bak TH, Bhatia KP, Borroni B, Boxer AL, Dickson DW, Grossman M, Hallett M, Josephs KA (2013). Criteria for the diagnosis of corticobasal degeneration. Neurology.

[9] Riley DE, Lang AE, Lewis AE, Resch L, Ashby P, Hornykiewicz O, Black S (1990). Cortical-basal ganglionic degeneration. Neurology.

[10] Boeve BF, Lang AE, Litvan I (2003). Corticobasal degeneration and its relationship to progressive supranuclear palsy and frontotemporal dementia. Annals of Neurology.

[11] Höglinger GU, Respondek G, Stamelou M, Kurz C, Josephs KA, Lang AE, Mollenhauer B, Müller U, Nilsson C, Whitwell JL, Arzberger T (2017). Clinical diagnosis of progressive supranuclear palsy: The movement disorder society criteria. Movement Disorders.

[12] Bak TH, Hodges JR (2008). Corticobasal degeneration: Clinical aspects. Handbook of Clinical Neurology.

[13] Mathew R, Bak TH, Hodges JR (2012). Diagnostic criteria for corticobasal syndrome: A comparative study. Journal of Neurology, Neurosurgery and Psychiatry.

[14] Lin YS, Lee WJ, Wang SJ, Fuh JL (2018). Levels of plasma neurofilament light chain and cognitive function in patients with Alzheimer or Parkinson disease. Scientific Reports.

[15] Aerts MB, Esselink RAJ, Bloem BR, Verbeek MM (2011). Cerebrospinal fluid tau and phosphorylated tau protein are elevated in corticobasal syndrome. Movement Disorders.

[16] Liberati A, Altman DG, Tetzlaff J, Mulrow C, Gøtzsche PC, Ioannidis JP, Clarke M, Devereaux PJ, Kleijnen J, Moher D (2009). The PRISMA statement for reporting systematic reviews and meta-analyses of studies that evaluate healthcare interventions: Explanation and elaboration. BMJ.

[17] Cumpston M, Li T, Page MJ, Chandler J, Welch VA, Higgins JP, Thomas J (2019). Updated guidance for trusted systematic reviews: A new edition of the cochrane handbook for systematic re-views of interventions. The Cochrane Database of Systematic Reviews.

[18] Ouzzani M, Hammady H, Fedorowicz Z, Elmagarmid A (2016). Rayyan: A web and mobile app for systematic reviews. Systematic Reviews.

[19] Lo CKL, Mertz D, Loeb M (2014). Newcastle-Ottawa Scale: Comparing reviewers’ to authors’ assessments. BMC Medical Research Methodology.

[20] Quadalti C, Calandra-Buonaura G, Baiardi S, Mastrangelo A, Rossi M, Zenesini C, Giannini G, Candelise N, Sambati L, Polischi B, Plazzi G (2021). Neurofilament light chain and α-synuclein RT-QuIC as differential diagnostic biomarkers in parkinsonisms and related syndromes. NPJ Parkinson's Disease.

[21] Morenas-Rodríguez E, Cervera-Carles L, Vilaplana E, Alcolea D, Carmona-Iragui M, Dols-Icardo O, Ribosa-Nogué R, Muñoz-Llahuna L, Sala I, Belen Sanchez-Saudinos M, Blesa R (2016). Progranulin protein levels in cerebrospinal fluid in primary neurodegenerative dementias. Journal of Alzheimer's Disease.

[22] Boman A, Svensson S, Boxer A, Rojas JC, Seeley WW, Karydas A, Miller B, Kågedal K, Svenningsson P (2016). Distinct lysosomal network protein profiles in parkinsonian syndrome cerebrospinal fluid. Journal of Parkinson's Disease.

[23] Doss S, Wandinger KP, Hyman BT, Panzer JA, Synofzik M, Dickerson B, Mollenhauer B, Scherzer CR, Ivinson AJ, Finke C, Schöls L (2014). High prevalence of NMDA receptor IgA/IgM antibodies in different dementia types. Annals of Clinical Translational Neurology.

[24] Meeter LH, Vijverberg EG, Del Campo M, Rozemuller AJ, Kaat LD, de Jong FJ, van der Flier WM, Teunissen CE, van Swieten JC, Pijnenburg YA (2018). Clinical value of neurofilament and phospho-tau/tau ratio in the frontotemporal dementia spectrum. Neurology.

[25] Delaby C, Alcolea D, Carmona-Iragui M, Illán-Gala I, Morenas-Rodríguez E, Barroeta I, Altuna M, Estellés T, Santos-Santos M, Turon-Sans J, Muñoz L (2020). Differential levels of neurofilament light protein in cerebrospinal fluid in patients with a wide range of neurodegenerative disorders. Scientific Reports.

[26] Borroni B, Premi E, Agosti C, Alberici A, Cerini C, Archetti S, Lanari A, Paghera B, Lucchini S, Caimi L, Padovani A (2011). CSF Alzheimer’s disease-like pattern in corticobasal syndrome: Evidence for a distinct disorder. Journal of Neurology, Neurosurgery and Psychiatry.

[27] Hansson O, Janelidze S, Hall S, Magdalinou N, Lees AJ, Andreasson U, Norgren N, Linder J, Forsgren L, Constantinescu R, Zetterberg H (2017). Blood-based NfL: A biomarker for differential diagnosis of parkinsonian disorder. Neurology.

[28] Hall S, Janelidze S, Zetterberg H, Brix B, Mattsson N, Surova Y, Blennow K, Hansson O (2020). Cerebrospinal fluid levels of neurogranin in Parkinsonian disorders. Movement Disorders.

[29] Alcolea D, Vilaplana E, Suárez-Calvet M, Illán-Gala I, Blesa R, Clarimón J, Lladó A, Sánchez-Valle R, Molinuevo JL, García-Ribas G, Compta Y (2017). CSF sAPPβ, YKL-40, and neurofilament light in frontotemporal lobar degeneration. Neurology.

[30] Schulz I, Kruse N, Gera RG, Kremer T, Cedarbaum J, Barbour R, Zago W, Schade S, Otte B, Bartl M, Hutten SJ (2021). Systematic assessment of 10 biomarker candidates focusing on α-synuclein-related disorders. Movement Disorders.

[31] Olsson B, Portelius E, Cullen NC, Sandelius Å, Zetterberg H, Andreasson U, Höglund K, Irwin DJ, Grossman M, Weintraub D, Chen-Plotkin A (2019). Association of cerebrospinal fluid neurofilament light protein levels with cognition in patients with dementia, motor neuron disease, and movement disorders. JAMA Neurology.

[32] Björkhem I, Patra K, Boxer AL, Svenningsson P (2018). 24S-Hydroxycholesterol correlates with tau and is increased in cerebrospinal fluid in Parkinson’s disease and corticobasal syndrome. Frontiers in Neurology.

[33] Benvenutto A, Guedj E, Felician O, Eusebio A, Azulay JP, Ceccaldi M, Koric L (2020). Clinical phenotypes in corticobasal syndrome with or without amyloidosis biomarkers. Journal of Alzheimer's Disease.

[34] Jabbari E, Holland N, Chelban V, Jones PS, Lamb R, Rawlinson C, Guo T, Costantini AA, Tan MM, Heslegrave AJ, Roncaroli F (2020). Diagnosis across the spectrum of progressive supranuclear palsy and corticobasal syndrome. JAMA Neurology.

[35] Luk C, Compta Y, Magdalinou N, Martí MJ, Hondhamuni G, Zetterberg H, Blennow K, Constantinescu R, Pijnenburg Y, Mollenhauer B, Trenkwalder C (2012). Development and assessment of sensitive immuno-PCR assays for the quantification of cerebrospinal fluid three- and four-repeat tau isoforms in tauopathies. Journal of Neurochemistry.

[36] Magdalinou NK, Paterson RW, Schott JM, Fox NC, Mummery C, Blennow K, Bhatia K, Morris HR, Giunti P, Warner TT, De Silva R (2015). A panel of nine cerebrospinal fluid biomarkers may identify patients with atypical parkinsonian syndromes. Journal of Neurology, Neurosurgery and Psychiatry.

[37] Borroni B, Malinverno M, Gardoni F, Grassi M, Parnetti L, Agosti C, Alberici A, Premi E, Bonuccelli U, Gasparotti R, Calabresi P (2010). A combination of CSF tau ratio and midsaggital midbrain-to-pons atrophy for the early diagnosis of progressive supranuclear palsy. Journal of Alzheimer's Disease.

[38] Hassan A, Whitwell JL, Josephs KA (2011). The corticobasal syndrome-Alzheimer’s disease conundrum. Expert Review of Neurotherapeutics.

[39] Ling H, Kovacs GG, Vonsattel JPG, Davey K, Mok KY, Hardy J, Morris HR, Warner TT, Holton JL, Revesz T (2016). Astrogliopathy predominates the earliest stage of corticobasal degeneration pathology. Brain.

[40] Kobylecki C, Mann DM (2016). Presymptomatic anterior frontal involvement in corticobasal degeneration. Brain.

[41] Josephs KA, Whitwell JL, Boeve BF, Knopman DS, Petersen RC, Hu WT, Parisi JE, Dickson DW, Jack CR (2010). Anatomical differences between CBS-corticobasal degeneration and CBS-Alzheimer’s disease. Movement Disorders.

[42] Shaw LM, Vanderstichele H, Knapik-Czajka M, Clark CM, Aisen PS, Petersen RC, Blennow K, Soares H, Simon A, Lewczuk P, Dean R (2009). Cerebrospinal fluid biomarker signature in Alzheimer’s disease neuroimaging initiative subjects. Annals of Neurology.

[43] Mattsson N, Zetterberg H, Janelidze S, Insel PS, Andreasson U, Stomrud E, Palmqvist S, Baker D, Hehir CAT, Jeromin A, Hanlon D (2016). Plasma tau in Alzheimer disease. Neurology.

[44] Llorens F, Villar-Piqué A, Candelise N, Ferrer I, Zerr I (2018). Tau protein as a biological fluid biomarker in neurodegenerative dementias. Cognitive Disorders.

[45] Zetterberg H, Skillbäck T, Mattsson N, Trojanowski JQ, Portelius E, Shaw LM, Weiner MW, Blennow K (2016). Association of cerebrospinal fluid neurofilament light concentration with Alzheimer disease progression. JAMA Neurology.

[46] Pillai JA, Bena J, Bebek G, Bekris LM, Bonner-Jackson A, Kou L, Pai A, Sørensen L, Neilsen M, Rao SM, Chance M (2020). Inflammatory pathway analytes predicting rapid cognitive decline in MCI stage of Alzheimer’s disease. Annals of Clinical Translational Neurology.

[47] Gaetani L, Blennow K, Calabresi P, Di Filippo M, Parnetti L, Zetterberg H (2019). Neurofilament light chain as a biomarker in neurological disorders. Journal of Neurology, Neurosurgery & Psychiatry.

[48] Mattsson N, Andreasson U, Zetterberg H, Blennow K (2017). Association of plasma neurofilament light with neurodegeneration in patients with Alzheimer disease. JAMA Neurology.

[49] Kuhle J, Disanto G, Lorscheider J, Stites T, Chen Y, Dahlke F, Francis G, Shrinivasan A, Radue EW, Giovannoni G, Kappos L (2015). Fingolimod and CSF neurofilament light chain levels in relapsing-remitting multiple sclerosis. Neurology.

[50] Miller TM, Cudkowicz ME, Genge A, Shaw PJ, Sobue G, Bucelli RC, Chiò A, Van Damme P, Ludolph AC, Glass JD, Andrews JA (2022). Trial of antisense oligonucleotide tofersen for SOD1 ALS. New England Journal of Medicine.

[51] Di Stefano F, Kas A, Habert MO, Decazes P, Lamari F, Lista S, Hampel H, Teichmann M (2016). The phenotypical core of Alzheimer’s disease-related and nonrelated variants of the corticobasal syndrome: A systematic clinical, neuropsychological, imaging, and biomarker study. Alzheimer's & Dementia.

[52] Constantinides VC, Paraskevas GP, Emmanouilidou E, Petropoulou O, Bougea A, Vekrellis K, Evdokimidis I, Stamboulis E, Kapaki E (2017). CSF biomarkers β-amyloid, tau proteins and a-synuclein in the differential diagnosis of Parkinson-plus syndromes. Journal of the Neurological Sciences.

